# Current breakthroughs and advances in atmospheric room temperature plasma (ARTP) technology for biomanufacturing

**DOI:** 10.1186/s40643-025-00907-3

**Published:** 2025-06-18

**Authors:** Yu-Hsiu Li, Jiun-Jang Juo, I-Son Ng

**Affiliations:** Department of Chemical Engineering, National Cheng Kung University, Tainan, 701 Taiwan

**Keywords:** Biomanufacturing, Atmospheric room temperature plasma, Yeast, Microalgae, High-value chemical

## Abstract

Atmospheric and Room Temperature Plasma (ARTP) mutagenesis has emerged as a novel and powerful physical mutation technology for microbial strain improvement recently. ARTP operates at atmospheric pressure and room temperature using a helium plasma jet, inducing widespread genomic mutations through reactive species and DNA damage. Compared to traditional mutagenesis methods, ARTP is safer, more efficient, and capable of producing high mutation rates without genetic modification, making it a valuable and sophisticated tool in biomanufacturing. This review outlines the principles and diverse applications of ARTP technology for enhancing enzyme activity, metabolite yields, and stress tolerance across various organisms. It also provides a comprehensive discussion of underlying biological mechanisms, workflow, optimization parameters, and potential cellular instability associated with ARTP-induced mutagenesis. Finally, current breakthroughs and future perspectives of ARTP mutagenesis are addressed, emphasizing its role in advancing next-generation microbial platforms for industrial biotechnology and environmental sustainability.

**Graphical Abstract:**

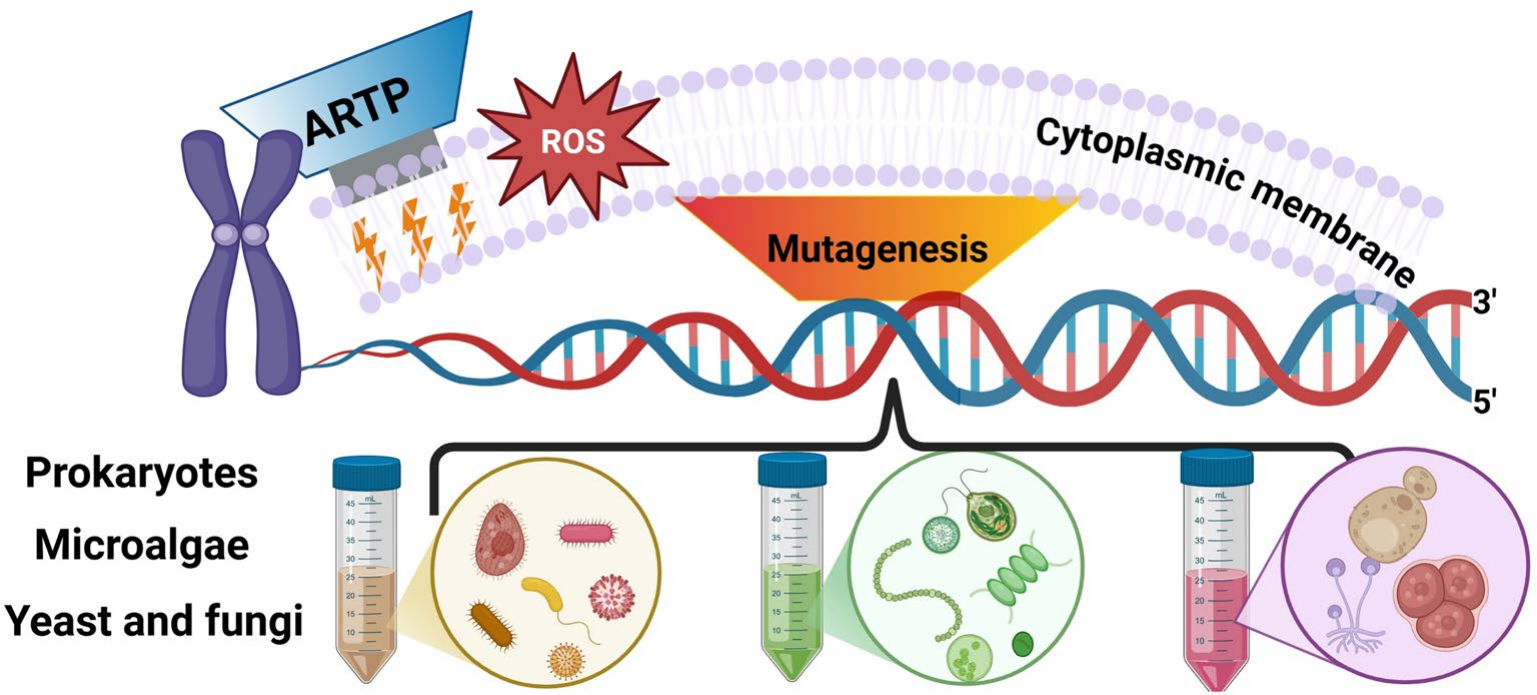

## Introduction

Microorganisms refer to microscopic life forms that are typically invisible to the naked eye, including archaea, bacteria, yeast, fungi, actinomycetes, protozoa, and algae. Owing to their small size, high surface-area-to-volume ratio, rapid metabolic rates, and strong adaptability to diverse environments, microorganisms are invaluable across many industrial sectors (Kochhar et al. 2022; Kuang et al. 2024). Microbial technologies have been widely applied in agriculture, petrochemicals, bioenergy, food production, pharmaceuticals, environmental remediation, and the breakdown of hazardous substances. All technologies not only enhance productivity but also promote sustainable development by minimizing environmental impact. As microbial biosynthesis of high-value chemicals has become more popular in the past 30 years, optimizing microbial performance is crucial for advancing biomanufacturing processes (Zhu et al. 2018).

Despite their advantages, microorganisms face metabolic bottlenecks (Glick et al. 1995; Pereira et al. 2018), limitations imposed by suboptimal growth conditions or toxic inhibitors (David et al. 2024) and reduce productivity due to genetic instability under stress during the continuous process. From an economic standpoint, the high costs associated with fermentation, product separation, and purification processes further restrict the large-scale commercialization of microbial-based systems. As a result, strain improvement has become a vital tool in unlocking the full industrial potential of microbial systems (Petrova et al. 2024).

Previous strategies have been developed for microbial strain improvement. In summary, the first choice is random mutagenesis and screening under physical, chemical or biological methods (Abaza et al. 2020; Cravens et al. 2021). Physical mutagenesis involves exposing cells to ultraviolet (UV) light, high-energy neutron or particle beams, X-rays, gamma rays, or ionizing radiation (Labadie et al. 2024; Zeng et al. 2017). Chemical mutagenesis employs alkylating agents, base analogs, ethyl methanesulfonate (EMS), nitrous acid, and sodium azide to induce random DNA damage (Auerbach et al. 1949; Kumar et al. 2015). Biological mutagenesis, on the other hand, relies on mechanisms such as adaptive laboratory evolution (ALE) (Wannier et al. 2018; Jiang et al. 2024), transposon insertion (Hoeller et al. 2008; Lu et al. 2022), or genetic material delivery via plasmids and viruses.

**Fig. 1:**
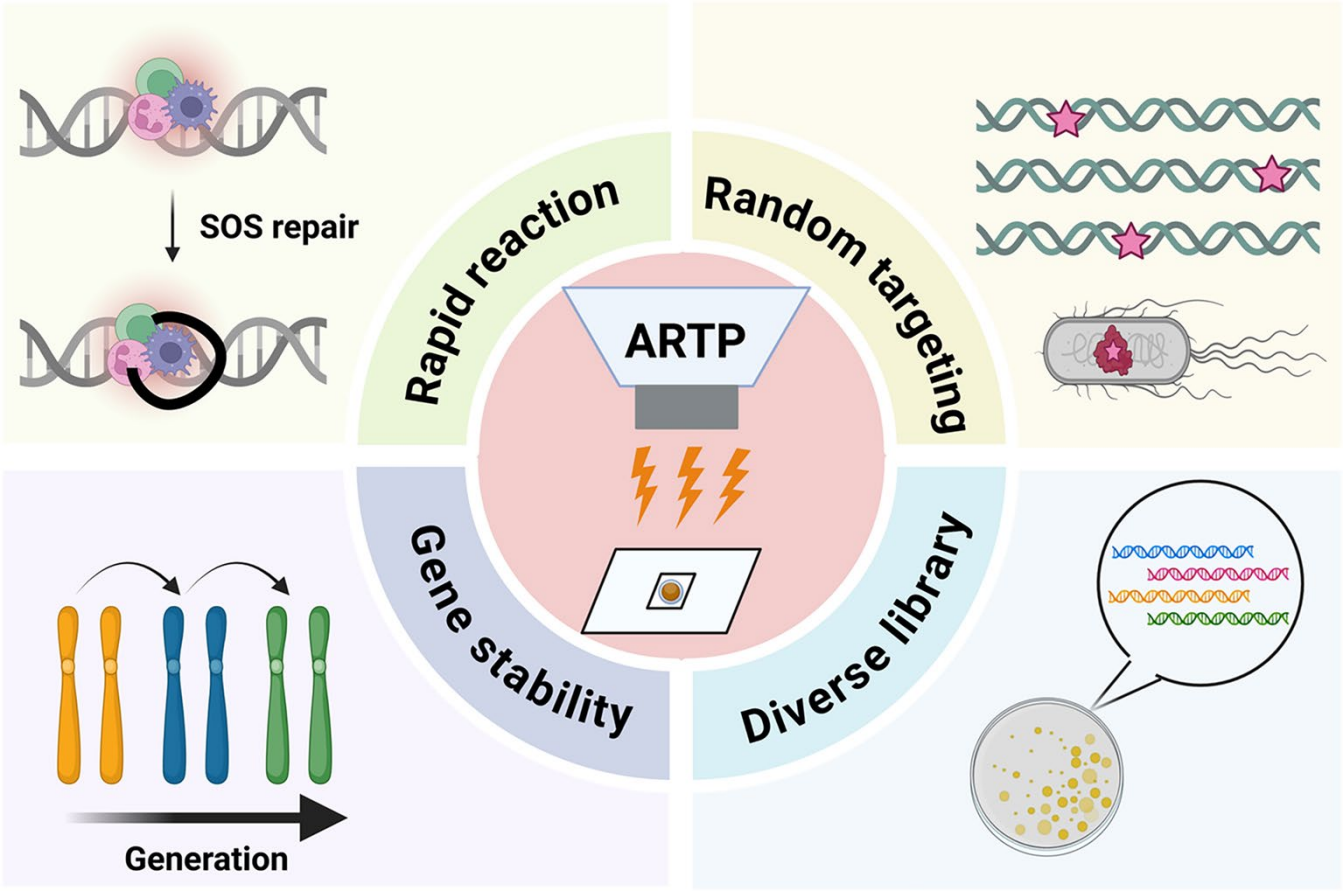
Advantages of ARTP mutagenesis in genetic engineering from rapid reaction, random targeting, diverse library and gene stability

**Fig. 2:**
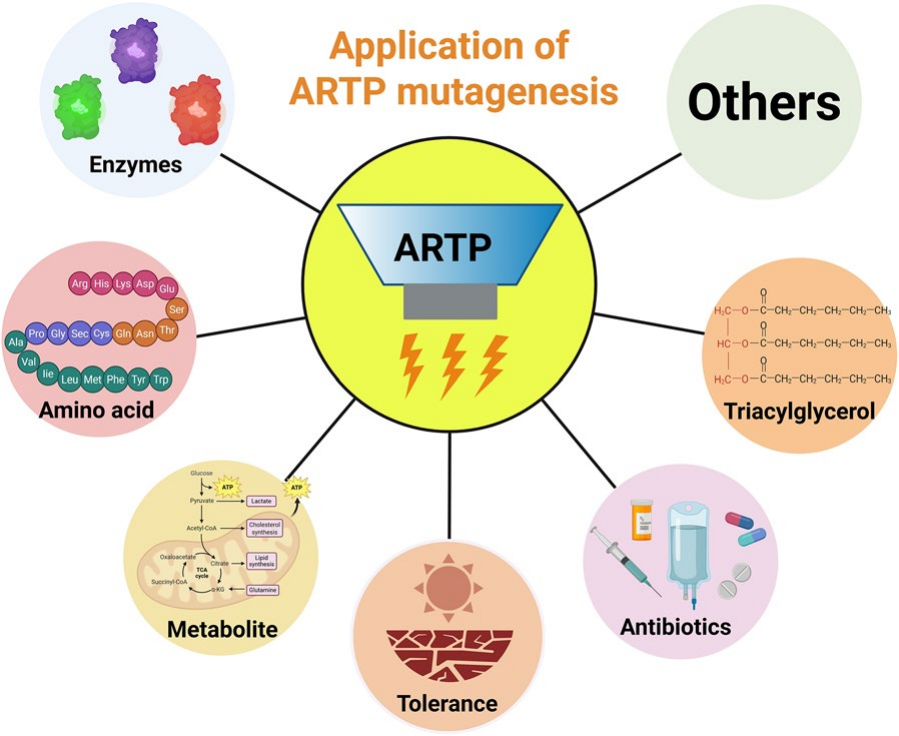
The Applications of ARTP mutagenesis in different research aspects, including enzymes and amino acid production, metabolite compounds, environmental issue, antibiotic and lipids or biofuel

Despite the widespread use of traditional mutagenesis techniques in microbial strain improvement, challenges such as operational complexity, low mutation rate, safety concerns, and limited social acceptance still persist. To overcome these problems, a novel physical mutagenesis technology, ARTP mutagenesis, has been developed in recent years. Unlike conventional approaches, ARTP has been shown to induce greater DNA damage and achieve higher mutation rates, as demonstrated through the integration of flow cytometry system for high-throughput screening (Zhang et al. 2014; Zhang et al. 2015). As shown in Table 1, ARTP offers rapid mutation speeds and generates large mutant library. Because it is a naturally random mutagenesis mechanism, it facilitates strain adaptation to extreme environments via self-induced mutations. Notably, ARTP also maintains high genetic stability, ensuring that beneficial mutations are preserved after serial passaging. (Fig. 1). This technology is developed by the Department of Chemical Engineering at Tsinghua University and commercialized in 2012 (Wu et al. 2017). Tt is now available in several commercial models, including ARTP-M, ARTP-IIS, ARTP-IIIS, and ARTP-A, which are design for use in various cell systems. ARTP mutagenesis employs radio-frequency energy to generate a non-thermal plasma jet under atmospheric pressure using high-purity helium gas. This plasma stream produces reactive species such as oxygen atoms, nitrogen radicals, ions, electrons, and hydroxyl radicals that cause DNA damage within microbial cells. The damage rapidly activates the SOS repair pathway (Maslowska et al. 2019), a mechanism that is inherently error-prone and introduces random mutations in different types of cells, including bacteria (Liu et al. 2020a), actinomycetes (Yu et al. 2022), fungi (Shu et al. 2020), yeasts (Zhong et al. 2024), and microalgae (Wang et al. 2024b). From the screening process, more production of enzymes, amino acids, secondary metabolites, lipids, and stress-resistant phenotypes are obtained (Fig. 2). Recently, ARTP has also been employed in microbial degradation of environmental pollutants, protein expression optimization, and the biosynthesis of pharmaceuticals and biofuels (Li et al. 2024b).

**Table 1:** Evaluation of the advantages for ARTP compared to other mutagenesis methods

Mutagenesis methods	Maximum SOS induction factor (Fi)	Proportion of cells with increased FIF* (%)	Mutation rate (X10^−9^ per mutations per generation)
4-NQO	1.50 ± 0.13	17.0	0.37
MNNG	1.58 ± 0.07	16.5	0.11
UV	1.66 ± 0.11	26.1	1.11
ARTP	2.79 ± 0.15	35.2	5.37

^*^FIF: Fluorescence intensity of fluorescein

In this review, we present a comprehensive overview of the principles and practical applications of ARTP technology in microbial strain development. The operational mechanisms and mutagenesis parameters of the ARTP system are demonstrated. We critically highlight the application of ARTP in prokaryotes, microalgae, yeasts, and fungi toward productivity and functionality. Finally, the prospective and limitations of this technology in industrial biotechnology are summarized. This review aims to support researchers and engineers in leveraging ARTP as a next-generation platform for microbial optimization in biomanufacturing.

## What is ARTP used for?

Before the development of ARTP technology, low-temperature plasma-based mutagenesis techniques were already in use. Among all, atmospheric pressure dielectric barrier discharge (AP-DBD) and atmospheric pressure radio frequency glow discharge (RF-APGD) were the most prominent (Tian et al. 2014; Farouk et al. 2008). AP-DBD utilizes various dielectric materials and high-voltage direct or alternating current to generate plasma, which directly interacts with biological samples. On the other hand, RF-APGD uses helium to generate a high concentration of chemically reactive species under uniform discharge and low-temperature conditions, which disrupt DNA and trigger microbial repair. ARTP system, developed from RF-APGD, has shown great potential for efficient microbial strain improvement (Wang et al. 2010; Li et al. 2012).

**Fig. 3:**
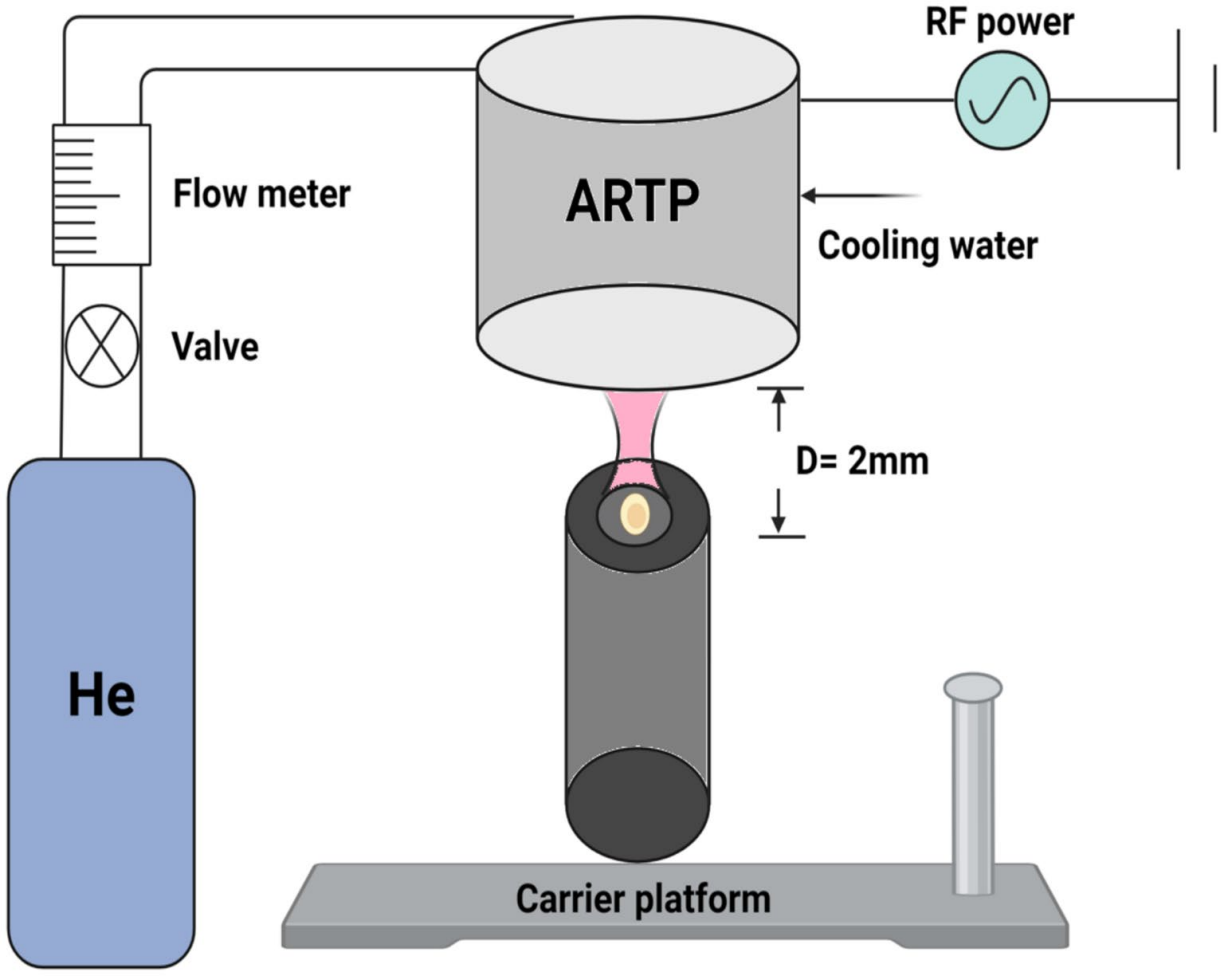
Scheme of atmospheric and room temperature plasma (ARTP) device. The carrier gas is helium and a high radiation frequency generator (RF) at 13.6 MHz are required in the system

ARTP mutagenesis is performed at room temperature and atmospheric pressure. It also uses high-purity helium gas (≥ 99.99%) as the plasma source (Fig. 3). When ionized by a radio-frequency electric field, helium generates a low-temperature plasma jet containing reactive oxygen and nitrogen species (RONS), including ions, electrons, radicals (e.g., OH), and excited atoms (Ottenheim et al. 2018; Liu et al. 2019). The reactive species interact with cellular DNA, proteins, and membranes, triggering oxidative stress and DNA damage. Interestingly, a study using artificially synthesized mononucleotides and oligonucleotides as models explored the molecular mechanisms induced by ARTP mutagenesis. Through nuclear magnetic resonance (NMR) spectroscopy, it was found that the structural changes in mononucleotides, such as dATP and dCTP, were more prone to cleavage. Furthermore, oligonucleotides also exhibited bond breakage upon ARTP treatment, with dT8 displaying the highest stability, maintaining a consistently high signal intensity. Therefore, when the DNA structure is damaged, cells activate the SOS repair pathway, which is error-prone and leads to the generation of random mutations (Wang et al. 2020a).

The mechanism of ARTP-induced mutagenesis is genome-wide, differing from site-specific gene editing. It induces base substitutions, deletions, insertions, and chromosomal rearrangements throughout the genome, allowing researchers to screen for mutants with enhanced traits, such as growth rate, metabolic output, or stress resistance (Zhang et al. 2023; Tian et al. 2020). Several parameters critically influence ARTP mutagenesis efficiency. For example, the irradiation power and exposure time affect the concentration of reactive species and the extent of DNA damage. Excessive power or duration can lead to high lethality and cell death, while insufficient exposure may fail to induce adequate mutations. A lethality rate of approximately 90% is generally optimal, as it ensures sufficient DNA disruption while retaining viable cells for screening (Li et al. 2008; Liu et al. 2023). On the other hand, the gas flow rate of helium is introduced to impact plasma density and reactive species concentration. A low flow rate may produce insufficient plasma, while a high rate can dissipate reactive species too quickly, reducing their contact with cells (Ottenheim et al. 2018). Typically, helium flow rates are set between 0 and 15 standard liters per minute (SLM). Finally, the distance between sample and plasma nozzle determined the energy delivered to the cells and the uniformity of plasma exposure. A standard distance of 2 mm is used to maintain reproducibility and ensure effective mutagenesis.

**Fig. 4:**
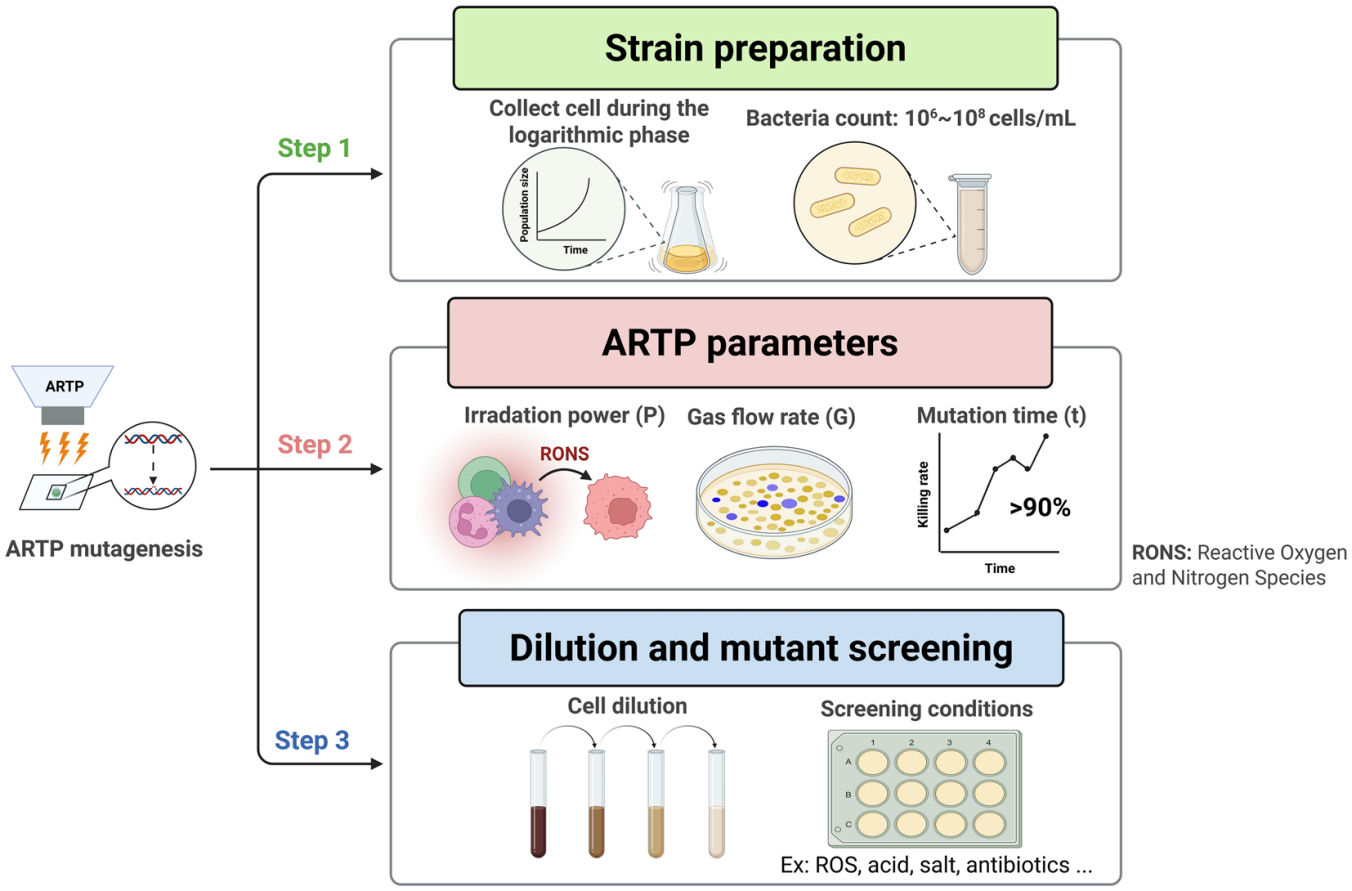
The key operations for ARTP mutagenesis process. Step 1: strain preparation tasks before mutagenesis; Step 2: includes power, glass flow rate and treatment time, which are the crucial parameters that affect the mutagenesis results; and Step 3: focus on screening the positive colony from liquid dilution

The ARTP mutagenesis workflow consists of three main stages: sample pretreatment, parameter optimization, and mutant screening (Fig. 4). At first, cells in the logarithmic growth phase are most suitable for ARTP exposure due to their high metabolic activity and sensitivity to external stimuli (Gott et al. 2019). The optimal cell concentration is typically an optical density (OD_600_) between 0.6 and 0.8 for prokaryotic cells. Before plasma treatment, cells are washed to remove metabolic residues and resuspended in fresh medium or sterilized water. A 10% glycerol solution is often added in a 1:1 ratio to enhance cell dispersion (Ma et al. 2020).

Key parameters shown in Table 2 demonstrated the power (typically 100–120 W), helium flow rate, and exposure time must be tailored to the organism. Prokaryotic species like bacteria typically require 15–120 s of exposure, while actinomycetes may need 30–180 s. Eukaryotic organisms, being more complex, generally require longer exposure, such as fungi (60–360 s), yeasts (30–240 s), and microalgae (5–150 s) (Ottenheim et al. 2018). After treatment, cells are serially diluted and plated to achieve optimal colony separation. An insufficient dilution may lead to dense colony growth, making it difficult to isolate single colony, whereas excessive dilution can result in too few colonies for effective screening. Once colonies are formed, an appropriate screening strategy can be applied to identify mutants with selection pressures. Screening conditions should reflect the desired phenotype, such as enhanced growth, product yield, or stress tolerance. Selective pressures may include high or low pH, temperature extremes, antibiotics, metal ions, or chemical agents. The next sections will detail recent breakthroughs in ARTP applications across different microbial taxa.

**Table 2:** General ARTP treatment time for samples

Mutagenesis strain types		General treatment time (s)
Prokaryotes	Bacteria	15–120
	Actinomycetes	30–180
Eukaryotes	Fungi	60–360
	Yeasts	30–240
	Microalgae	5–150

^*^ARTP parameters used 120 W, 10 SLM, and 2 mm

## The current breakthrough by using ARTP

### Mechanism and application of ARTP in prokaryotes

In bacteria, gram-positive bacteria do not have an outer membrane and a relatively thick cell wall, with only the reactive species generated by the plasma interacting with the phospholipid bilayer (PLB) of the cytoplasmic membrane, causing lipid peroxidation reactions. In contrast, gram-negative bacteria have an outer membrane rich in lipopolysaccharides (LPS). Due to the strong negative charge of LPS, positively charged particles from the plasma are attracted to the bacterial surface and cause membrane damage. Therefore, gram-positive bacteria are generally more resistant to high-power output or prolonged plasma exposure (Martines et al. 2020). This phenomenon is also observed in the studies conducted by Montie et al. (2000) and Lunov et al. (2016). Table 3 summarizes the parameters and achievements of various bacteria under ARTP mutagenesis.

**Table 3:** Application of ARTP mutagenesis technology in prokaryotes over the past five years

Optimal strain	ARTP parameters				Improvement (%)	Ref
	P (W)	G (SLM)	D (mm)	T (s)		
*Actinobacillus succinogenes M4*	120	10	2	120	113% succinic acid	Wu et al. (2024)
*Bacillus coagulans BC15*	120	8	2	15	22.4% survival rate	Liu et al. (2020a)
*Bacillus subtilis A59*	120	8	2	15	23.38% alkaline protease activity	Liu et al. (2020b)
*Chryseobacterium proteolyticum WG15*	100	10	2	30	48.69% protein glutaminase	Wang et al. (2023)
*Corynebacterium glutamicum MA6*	100	10	2	120	91% VHH yield	Meng et al. (2021)
*Escherichia coli 60AP03*	–	–	–	150	120% L-cysteine	Yang et al. (2024b)
*Escherichia coli C5*	120	10	2	120	41.77% L-tryptophan	Ye et al. (2024)
*Escherichia coli GS-2–4*	100	10	2	-	37% 4HPAA production	Shen et al. (2022)
*Escherichia coli GS-2–7*	100	10	2	-	170% shikimic acid	Niu et al. (2020)
*Escherichia coli KP-FMME-6*	100	10	2	-	23.5% 1,3-propanediol	Zhang et al. (2024)
*Exiguobacterium profundum 10,017*	100	10	2	120	20% protease activity	Xin et al. (2020)
*Hyphomicrobium denitrifcans FJAU-A26*	100	10	2	60	148% PQQ	Ren et al. (2023)
*Pseudomonas putida NB10*	100	10	2	20	100% BDO consumption	Qian et al. (2023)
*Rhodobacter sphaeroides R.S 17*	100	10	2	30	22.1% CoQ10 productivity	Wang et al. (2021b)
*Streptococcus zooepidemicus SFPE-A17*	100	10	2	150	42.9% hyaluronan	Yao et al. (2023)
*Streptomyces fradiae Sf 6–2*	40	10	2	180	100% neomycin sulfate	Yu et al. (2022)
*Streptomyces natalensis DES-26*	100	10	2	40	86.36% natamycin	Sun et al. (2023)
*Streptomyces rimosus M527-pAN-S38*	100	10	2	–	52% rimocidin	Jiang et al. (2022)
*Streptomyces roseosporus L2201*	100	10	3	150	58.33% daptomycin	Zhu et al. (2023)

#### Enzyme activity and protein production

The application of ARTP in enhancing enzyme activity has been demonstrated in various studies. Xin et al. (2020) applied ARTP to enhance the extraction of chitin from shrimp waste. By evaluating the protease activity and deproteinization rate (DP%) of mutant library, the resulting mutant strain MS 10017 exhibited significantly improved protease activity and deproteinization efficiency, with increases of 36.39% and 11.9%, respectively. Liu et al. (2020b) developed the protease-producing mutant strain A59 through ARTP mutagenesis, resulting in a 23.38% increase in enzyme activity. In another study, Meng et al. (2021) applied ARTP in combination with high-throughput screening to boost the production of the therapeutic protein VHH in *Corynebacterium glutamicum*. The resulting mutant strain MA6 not only exhibited a 91% increase in protein yield but also demonstrated excellent growth characteristics and fermentation stability. Similarity, Wang et al. (2021b) applied ARTP under multiple screening pressures to improve coenzyme Q10 production from *Rhodobacter sphaeroides*, achieving a 22.1% increase in yield. When combined ARTP with adaptive laboratory evolution (ALE) and methanol pressure selection, it is feasible to obtain the best mutant with high levels of pyrroloquinoline quinone (PQQ) (Ren et al. 2023). The resulting strain FJNU-A26 exhibited a 148% increase in PQQ yield. By using different screening strategy, Wang et al. (2023) employed ARTP and lithium chloride co-mutagenesis to generate strain WG15, which showed a 48.69% increase in protein glutaminase (PG) production under malonic acid screening.

#### High-value chemical production

ARTP has also been pivotal in boosting the synthesis of industrially relevant chemicals. By developing a biosensor-assisted ARTP, hyaluronic acid (HA) production was improved using the mutant SFPE-A17 with a 42.9% yield increase (Yao et al. 2023). Transcriptomic analysis indicated that the downregulation of cell wall synthesis genes redirected metabolic flux toward HA biosynthesis. Same idea was applied to evolve shikimic acid production (Niu et al. 2020).

On the other hand, ARTP mutant exhibited a twofold increase in stress tolerance, which included enhanced resistance to inhibitors derived from lignocellulosic hydrolysate, along with a 113% rise in succinic acid (SA) production compared to the wild type (Wu et al. 2024). One significant breakthrough was presented in an osmotolerant mutant for 1,3-propanediol production, that achieved a final titer of 118 g/L after further pathway engineering and gene knockouts (Zhang et al. 2024). Recently, Shen et al. (Shen et al. 2022) leveraged biosensor-assisted screening and genome shuffling on an ARTP-generated mutant library to develop *E. coli* strain GS-2–4, which yielded a 37% increase in 4-hydroxyphenylacetic acid (4HPAA). To date, biosensor-guided ARTP mutagenesis represents the most effective approach for obtaining non-random, targeted mutants.

#### Amino acid biosynthesis

ARTP has shown great potential in optimizing amino acid production. Ye et al. (2024) utilized ARTP and biosensor screening to obtain a mutant with a 41.77% increase in L-tryptophan production. Critically, L-cysteine production presents unique challenges due to its strong reducing nature, which generates reactive oxygen species (ROS) and DNA damage. Yang et al. (2024b) addressed this by combining ARTP and ALE to develop strains with increased L-cysteine tolerance. By overexpressing the *cys*E gene in the stabilized mutant, they achieved a 2.2-fold increase in L-cysteine concentration.

#### Antibiotic production and resistance

In antibiotic biosynthesis, ARTP has enabled significant improvements in product yield and resistance. Yu et al. (2022) increased neomycin potency by 100% in *Streptomyces fradiae* through ARTP mutagenesis and fermentation optimization. Sun et al. (2023) employed a combined UV–ARTP–DES mutagenesis strategy, resulting in an 86.36% increase in natamycin production in strain DES-26. Jiang et al. (2022) enhanced rimocidin production using reporter-guided selection and ARTP, increasing output by 52% and boosting gene expression of both *rim* and *neo* markers. Zhu et al. (Zhu et al. 2023) also used a dual-reporter system and ARTP mutagenesis to increase daptomycin production by 58.33%, suggesting the powerful results to increase different antibiotic production in the future.

#### Environmental applications and tolerance engineering

Beyond biosynthesis, ARTP has been instrumental in environmental applications such as plastic degradation. Qian et al. (2023) used ARTP and ALE to develop a *Pseudomonas putida* strain with improved tolerance to toxic intermediates during 1,4-butanediol degradation. This strain showed strong potential for plastic waste recycling. Liu et al. (2020a) developed a *Bacillus coagulans* mutant with enhanced bile salt tolerance using ARTP and ALE. The strain demonstrated 22.4% survival under 0.3% bile salt, with reduced membrane permeability and increased cell surface hydrophobicity and traits favorable for probiotic applications. Similarly, Cui et al. (2018) successfully developed a methanol-tolerant mutant strain of *Methylobacterium extorquens* AM1 through a strategy combining ARTP with ALE. This strain demonstrated a 610% increase in cell density under 5% (v/v) methanol. Both of them suggest that ALE is a powerful tool to complement ARTP in obtaining strains with enhanced phenotypic traits. Furthermore, next-generation sequencing (NGS) revealed that organic solvent tolerance is a complex phenotype governed by multiple genes.

In other representative study, Lu et al. (2011) demonstrated that ARTP mutagenesis promoted mutant growth and enhanced hydrogen production via the NADH pathway by increasing ATP yield and maintaining a low oxidation state balance. These findings confirm that the metabolic pathways in the mutants were altered. Similarly, Meng et al. (2024) introduced ARTP mutants into the *Apis mellifera*, and found that a point mutation in the *mglB* gene changed colony morphology and affected genetic variation in the mutual gliding locus, enhancing colonization efficiency in the non-native host.

### Application of ARTP in microalgae

Such as lipids, carotenoids (e.g., astaxanthin), polyunsaturated fatty acids (e.g., eicosatetraenoic acid [EPA] and docosahexaenoic acid [DHA]), and proteins. Table 4 summarizes representative applications of ARTP mutagenesis in microalgae.

**Table 4:** Application of ARTP mutagenesis technology in microalgae over the past five years

Optimal strain	ARTP parameters				Improvement (%)	Refs
	P (W)	G (SLM)	D (mm)	T (s)		
*Aurantiochytrium sp. R2A35*	120	10	2	60	14.3% DHA content	Wang et al. (2024b)
*Auxenochlorella pyrenoidosa A4-1*	120	10	2	15	31% protein content	Liu et al. (2023)
*Auxenochlorella pyrenoidosa MMC-8*	120	10	2	15	40.11% protein	Liu et al. (2024)
*Chlamydomonas reinhardtii A7S80*	120	10	2	45	Decrease 99.22% chlorophyll content	Cao et al. (2024)
*Crypthecodinium cohnii 16D*	150	10	2	40	130% DHA yield	Lv et al. (2020)
*Desmodesmus sp. AT151*	120	5	2	65	10.71% lipid 69.04% biomass	Li et al. (2023)
*Desmodesmus sp. AT60-15*	120	5	2	65	134% triglyceride	Sun et al. (2020)
*Desmodesmus sp. B15*	120	5	2	90	48.98% triglyceride 114.99% lipid content	Sun et al. (2022)
*Gracilariopsis lemaneiformis HAGL-X5*	120	10	2	46	50% of agar content	Xiao et al. (2023)
*Haematococcus lacustris 110–2*	120	5	2	40	43% astaxanthin 51% lipid	Ma et al. (2024)
*Parachlorella kessleri M8*	100	10	2	40	44% lipid productivity	Elshobary et al. (2022)
*Schizochytrium limacinum LD11*	120	10	2	20	12.04% lipid content 25.51% DHA yield	Chen et al. (2022)
*Schizochytrium sp. 6–23*	100	10	2	60	55.3% DHA	Zeng et al. (2021)
*Schizochytrium sp. A-32*	100	10	2	50	82% EPA	Ou et al. (2023)
*Schizochytrium sp. A78*	120	10	2	40	54.1% DHA content	Liu et al. (2021a)
*Ulothrix A20*	120	5	2	60	111.96% lipid	Yin et al. (2024)

#### Lipid and triacylglycerol production

Sun et al. (2020) applied ARTP to *Desmodesmus sp.*, screening for mutants with high biomass and triacylglycerol (TAG) content. The selected strain exhibited elevated expression of key lipid biosynthesis enzymes, leading to enhanced TAG accumulation. Similarly, ARTP on marine microalga *Parachlorella kessleri* evolved a mutant M8, which had 44% increase in lipid productivity (Elshobary et al. 2022). Notably, mutant strain showed the highest levels of saturated fatty acids and the lowest levels of polyunsaturated fatty acids, characteristics desirable for biodiesel production.

Yin et al. (2024) applied ARTP mutagenesis to the non-oleaginous green microalga *Ulothrix SDJZ-17* and obtained the mutant strain A20 with enhanced CO_2_ tolerance and lipid accumulation. Under 15% CO_2_ (v/v), A20 exhibited significantly improved growth and photosynthetic performance, reaching a light conversion efficiency (LCE) of 14.79% and a lipid content of 22.45% compared to wild-type, making it a promising candidate for biofuel applications under elevated CO_2_ conditions. Through multiple rounds of screening from the ARTP mutant library based on high lipid fluorescence intensity and growth rate, the dominant mutant strain AT151 was successfully isolated. This strain exhibited a 69.04% increase in lipid yield and formed a symbiotic relationship with other dominant indigenous strains during cultivation, demonstrating the potential applicability of ARTP in wastewater-based circular bioprocesses (Li et al. 2023). In another study, malonic acid was used as a novel selective pressure during ARTP mutagenesis. As a result, the strain B15 exhibited a 48.98% increase in TAG and a 114.99% increase in total lipid content (Sun et al. 2022). Upregulation of key genes involved in lipid metabolism was confirmed, supporting the strain’s potential for industrial-scale lipid production.

#### Omega-3 fatty acids: EPA and DHA

*Schizochytrium sp.* as the outstanding starting species was mutated using ARTP with chemical reagent stress screening to enhance EPA biosynthesis. The best mutant showed an 82% increase in EPA production compared to the wild type (Ou et al. 2023). In the context of DHA synthesis, Ma et al. (2024) co-applied ARTP and ethanol stress on *Haematococcus lacustris*, resulting in the mutant strain 110–2. This strain demonstrated a 43% increase in astaxanthin and a 51% increase in lipid content, with transcriptomic analysis confirming upregulation of lipid and carotenoid biosynthesis pathways. Lv et al. (2020) employed iodine vapor-based high-throughput screening and ARTP to develop strain 16D, a starch-deficient mutant of *Crypthecodinium cohnii*. This strain showed a 1.3-fold increase in DHA yield and a 1.7-fold increase in productivity. Metabolomic analysis revealed increased intracellular tagatose abundance, which may support enhanced lipid biosynthesis. Similarly, many studies have applied ARTP along with iodine acetate and clethodim inhibitors, respectively, to obtain high-DHA-producing *Schizochytrium* strains. Both approaches successfully addressed the limitations of traditional breeding methods and increased EPA or DHA yields (Zeng et al. 2021; Liu et al. 2021a; Chen et al. 2022; Wang et al. 2024b).

#### Protein, amino acid and others

ARTP mutagenesis has also been used to improve protein production in microalgae. Liu et al. (2023) developed a chlorophyll-deficient mutant strain A4-1 of *Auxenochlorella pyrenoidosa* using ARTP. This strain showed 31% higher protein content and 22% more total amino acids. The following year, the same team (Liu et al. 2024) integrated ARTP with high-throughput microdroplet culture (MMC) to rapidly screen for high-protein mutants, achieving a 40.11% increase in protein content under heterotrophic conditions. Similarly, Cao et al. (2024) applied ARTP to *Chlamydomonas reinhardtii* and obtained a mutant with a 99.22% reduction in chlorophyll content, making it suitable for food and feed applications due to improved sensory properties and digestibility.

For other screening methods in microalgae, Fang et al. (2013) successfully developed a dilution method in which individual filaments were separated in 96-well microplates, replacing the traditional colony phenotypes screening. This method efficiently isolates the mutants with distinct phenotypes. Furthermore, it demonstrated the potential applicability of this approach for screening mutants with enhanced tolerance to various environmental stresses. Xiao et al. (2023) applied ARTP and high-salt screening to breed *Gracilariopsis lemaneiformis* mutants with enhanced osmotic stress tolerance. The resulting strains not only adapted to saline conditions but also showed a 50% increase in agar yield, supporting their industrial relevance.

### Application of ARTP in yeasts and fungi

To improve yeast and fungal strains for industrial fermentation of pharmaceuticals, and food, ARTP induced genome-wide mutations without introducing foreign DNA, allowing for safe and efficient strain enhancement. The specific results are documented in carotenoids, alcohols, polysaccharides, organic acids and aromatics compounds in the following.

#### Carotenoids and alcohols production

Wang et al. (2020b) utilized ARTP to generate the mutant strain WY-239 of *Blakeslea trispora*, which exhibited a 56.27% increase in lycopene production. Table 5 illustrates this increase in lycopene production in the WY-239 mutant strain. The strain was initially identified through screening on solid medium and further confirmed by transcriptomic analysis. To address the challenge of balancing xylose utilization and inhibitor tolerance in yeast, Wei et al. (2020) performed multiple rounds of ARTP mutagenesis on *Saccharomyces cerevisiae*. The final mutant, 6 M-15, not only restored xylose consumption efficiency but also improved ethanol production by 15%.

**Table 5:** Application of ARTP mutagenesis technology in yeasts and fungi over the past five years

Optimal strain	ARTP parameters				Improvement (%)	Refs
	P (W)	G (SLM)	D (mm)	T (s)		
*Aspergillus niger AT 24*	120	10	2	120	36.5% citrate yield	Wang et al. (2021a)
*Aspergillus oryzae B-2*	120	10	2	150	8.5% protease	Shu et al. (2020)
*Blakeslea trispora WY-239*	150	10	2	150	56.27% lycopene	Wang et al. (2020b)
*Cyberlindnera jadinii WB15*	120	10	2	30	40% ribonucleic acid content	Li et al. (2024a)
*Fusarium fujikuroi 3–6-1*	100	–	–	70	13.86% gibberellic acid	Li et al. (2022)
*Grifola frondosa GFA2*	120	10	2	60	9.2% polysaccharide	Liu et al. (2021b)
*Inonotus obliquus A27*	120	10	2	140	137.67% polysaccharide	Hua et al. (2024)
*Komagataella phaffii Δmyo1-CH2*	120	10	2	120	36.8% hLYZ bioactivity	Zhong et al. (2024)
*Myrothecium verrucaria 3H6*	100	10	2	85	106.15% oxidase	Gou et al. (2022)
*Penicillium camembertii P12*	100	10	2	150	800% lipase activity	Ji et al. (2025)
*Phaffia rhodozyma Y1*	120	12	2	50	21.2% carotenoid content	Zhuang et al. (2020)
*Pleurotus djamor 240S-4*	100	10	2	240	28% protein	Pan et al. (2024)
*Saccharomyces boulardii MSBV*	100	12	5	40	56.77% selenium yield	Nie et al. (2022)
*Saccharomyces cerevisiae 6 M-15*	120	10	2	15	15% ethanol productivity	Wei et al. (2020)
*Saccharomyces cerevisiae HF-130*	120	10	2	35	98.8% ethanol	Hong et al. (2023)
*Saccharomyces cerevisiae NK-M21*	115	10	2	90	660% *p*-coumaric acid	Cai et al. (2021)
*Saccharomyces cerevisiae T11-1*	140	10	2	180	142.86% SAM	Weng et al. (2022)
*Yarrowia lipolytica A4*	120	10	2	420	28.9% pyruvic acid	Yuan et al. (2020)
*Yarrowia lipolytica M2*	120	10	2	60, 65	160% astaxanthin	Wang et al. (2024a)

Yuan et al. (2020) cultivated a salt-tolerant *Yarrowia lipolytica* mutant strain A4 using ARTP under high-salt conditions. This strain achieved a 28.9% increase in pyruvic acid yield. *Saccharomyces cerevisiae* strain HF-130 was developed after mutation, which showed a 98.8% increase in ethanol output under ultra-high concentration fermentation (Hong et al. 2023). In this study, enhanced ester production was also achieved by gene overexpression, resulting in a 130% rise in ethyl acetate content. More recently, using sequential ARTP and UV mutagenesis to generate the mutant Y1 of *Phaffia rhodozyma* can yield 21.2% more carotenoids under diphenylamine selection. Furthermore, by using ultrasound and cellulase treatments, a 96.01% astaxanthin extraction rate was also achieved (Zhuang et al. 2020).

#### Polysaccharide and organic acid

ARTP has also proven effective for enhancing polysaccharide biosynthesis. The growth rate and polysaccharide yield of *Grifola frondosa* has successfully changed in monosaccharide composition and superior productivity after ARTP (Liu et al. 2021b), while using *Inonotus obliquus* protoplasts, a mutant with a remarkable 137.67% increase in polysaccharide yield was observed (Hua et al. 2024).

In the domain of organic acid production, Wang et al. (2021a) used ARTP to generate *Aspergillus niger* mutant strain AT 24. This strain showed a 36.5% increase in citric acid output and, when combined with a co-saccharification strategy, achieved a 35.8% boost in fermentation efficiency. On the other hand, Li and his colleagues also used ARTP combined with ketoconazole-based screening to generate *Fusarium fujikuroi* mutant strain 3–6-1 (Li et al. 2022). This strain showed a 2.5-fold increase in gibberellic acid (GA3) production compared to the original strain. In addition, when combined with fed-batch fermentation, the GA3 production was further enhanced by 13.86%. Both studies demonstrated the effectiveness of ARTP in improving strain capabilities for enhanced organic acid production.

#### Aromatic and functional compounds

To enhance p-coumaric acid biosynthesis, Cai et al. (2021) knocked out the HTZ1 gene in *S. cerevisiae* and applied ARTP for further improvement. The resulting mutant NK-M21 produced 660% more p-coumaric acid, attributed to improved tyrosine pathway flux. In selenium-enriched yeast production, chemical Na₂SeO₃ was introduced as a selective pressure in ARTP mutagenesis to successfully develop *S. boulardii* mutant MSBV, which increased selenium accumulation by 56.8% (Nie et al. 2022).

For high value astaxanthin biosynthesis, Wang et al. (2024a) conducted ARTP mutagenesis on *Yarrowia lipolytica*, resulting in mutant strain M2. Transcriptome analysis revealed that redirected metabolic flux was upregulating in an alternative pathway and inhibited the central carbon metabolism, thus increased astaxanthin production.

#### Cofactors, RNA, and proteins

S-adenosyl-L-methionine (SAM) is a crucial methyl donor involved in various biochemical reactions. Weng et al. (2022) applied a combination of ARTP, UV, and LiCl mutagenesis with ALE using droplet microfluidics. The resulting strain T11-1 showed a 142.86% increase in SAM production. Transcriptomic data suggested upregulation of the TCA cycle and glycolysis pathways, along with reduced serine and ergosterol biosynthesis. Li et al. (2024a) applied ARTP to *Cyberlindnera jadinii* and obtained mutant WB15, which showed a 40% increase in RNA content. After optimizing the fermentation medium, RNA content further rose by 48.9%.

In protein production, Shu et al. (2020) used ARTP mutagenesis to isolate an *Aspergillus oryzae* strain with elevated acid protease activity, yielding an 8.5% improvement. Gou et al. (2022) enhanced acid dye oxidase production in *Myrothecium* using ARTP and UV, achieving increases of 106.15% and 97%, respectively. These enzymes hold potential for eco-friendly dye removal in wastewater treatment. Pan et al. (2024) optimized mycelial protein production in *Pleurotus djamor* using ARTP and hybridization, isolating mutant strain 240S-4, which exhibited a 28% increase in protein yield. Zhong et al. (2024) applied ARTP to *Komagataella phaffii* and, through transcriptome analysis, linked increased recombinant lysozyme (hLYZ) production to the downregulation of core cell division proteins. Gene knockout further validated these findings in fed-batch fermentation. Ji et al. (2025) used methanol stress and ARTP to generate *Penicillium camembertii* mutant P12, which demonstrated an 800% increase in lipase activity. The mutant also exhibited higher thermal and methanol stability and was capable of catalyzing the production of 1,3-diacylglycerol with a yield of 74.7%.

## Prospective and limitation

As aforementioned, the advancement of ARTP mutagenesis technology represents a significant breakthrough in microbial strain development. Especially it is a high-efficiency, non-GMO (Twardowski and Małyska, 2015), and environmentally friendly method, thus ARTP has attracted growing attention in both academic research and industrial applications. Looking forward, several key prospects and challenges will shape its future trajectory. From a technological standpoint, ARTP mutagenesis enables rapid and reproducible generation of diverse mutant libraries under atmospheric pressure and room temperature conditions. By integrating ARTP with high-throughput screening platforms, such as microdroplet culture systems and biosensor-guided selection, the discovery of high-performing strains can be significantly accelerated.

In recent years, the integration of ARTP with complementary mutagenesis and evolution strategies has further enhanced its utility. For instance, combining ARTP with ultraviolet (UV) irradiation (Yu et al. 2024), chemical mutagens such as diethyl sulfate (DES) (Sun et al. 2023), or adaptive laboratory evolution (ALE) (Liu et al. 2020a) have been shown to improve mutation diversity, stability, and trait optimization. Additionally, the incorporation of selective pressures, such as temperature extremes, pH fluctuations, osmotic stress, toxic compounds, or nutrient limitations, can guide the evolution of strains with specialized phenotypic traits.

Another promising direction involves the application of ARTP mutagenesis in environmental biotechnology. ARTP-induced mutants with enhanced capabilities for biodegradation and bioconversion can be employed to remediate pollutants or convert waste into valuable bio-based products. For example, engineered strains with improved tolerance to cytotoxic intermediates or enhanced metabolic pathways can be applied in plastic degradation, wastewater treatment, and heavy metal recovery.

Whole-genome sequencing (WGS) and transcriptomic analyses have begun to play a critical role in deciphering the mutational landscape introduced by ARTP. These tools facilitate the identification of key mutations responsible for enhanced traits, offering deeper insight into the genetic and regulatory mechanisms involved (Yang et al. 2024a). Such knowledge enables targeted metabolic engineering and rational design of second-generation mutants, further extending the application of ARTP in synthetic biology and systems biotechnology.

Despite its potential, ARTP technology has limitations. Its random mutagenesis leads to high variability, requiring large-scale screening. It lacks single-nucleotide precision, limiting fine-tuning of metabolic pathways without complementary tools. Additionally, ARTP parameters still need case-specific optimization. Moreover, the instability of cell behavior must be monitored for a while. Finally, the scale-up of ARTP from laboratory to industrial settings is still in its early stages. Current commercial ARTP systems are primarily designed for small-volume screening. For broader industrial adoption, engineering solutions to increase throughput, process automation, and real-time monitoring will be essential.

## Conclusion remarks

ARTP mutagenesis is a powerful tool for microbial biotechnology by enabling broad-spectrum genomic diversification without reliance on genetic modification. Unlike conventional approaches, ARTP balances efficiency, safety, and flexibility, making it uniquely suited for the rapid development of high performance strains across diverse taxa. It provides a new pathway for strain engineering, particularly in cases where traditional rational design is constrained by limited genomic insight. However, unlocking the full potential of ARTP demands a deeper understanding of its mutational dynamics and more sophisticated integration with genome-scale analytical platforms. While not a standalone solution, ARTP serves as a catalytic enabler for synthetic biology and next generation biomanufacturing, especially when rational design is limited. Further insight into its mutational dynamics and integration with genome-scale tools will enhance its full potential.

## Data Availability

The datasets used and/or analyzed during the current study are available from the corresponding author on reasonable request.
